# Bibliometric Analysis of Chimeric Antigen Receptor-Based Immunotherapy in Cancers From 2001 to 2021

**DOI:** 10.3389/fimmu.2022.822004

**Published:** 2022-03-30

**Authors:** Zhanpeng Ou, Ling Qiu, Haixu Rong, Bowen Li, Siqi Ren, Shijia Kuang, Tianjun Lan, Hsinyu Lin, Qunxing Li, Fan Wu, Tingting Cai, Lingjian Yan, Yushan Ye, Song Fan, Jinsong Li

**Affiliations:** ^1^ Guangdong Provincial Key Laboratory of Malignant Tumor Epigenetics and Gene Regulation, Sun Yat-sen Memorial Hospital, Guangzhou, China; ^2^ Department of Oral and Maxillofacial Surgery, Sun Yat-sen Memorial Hospital, Sun Yat-sen University, Guangzhou, China

**Keywords:** chimeric antigen receptors (CARs), CAR-based immunotherapy, bibliometric analysis, solid tumors, natural killer cells, safety and toxicity, gene editing

## Abstract

**Background:**

Chimeric antigen receptor (CAR)-based immunotherapy has shown great potential for the treatment of both hematopoietic malignancies and solid tumors. Nevertheless, multiple obstacles still block the development of CAR-based immunotherapy in the clinical setting. In this study, we aimed to summarize the research landscape and highlight the front lines and trends of this field.

**Methods:**

Literature published from 2001 to 2021 was searched in the Web of Science Core Collection database. Full records and cited references of all the documents were extracted and screened. Bibliometric analysis and visualization were conducted using CiteSpace, Microsoft Excel 2019, VOSviewer and R software.

**Results:**

A total of 5981 articles and reviews were included. The publication and citation results exhibited increasing trends in the last 20 years. *Frontiers in Immunology* and *Blood* were the most productive and most co-cited journals, respectively. The United States was the country with the most productive organizations and publications in the comprehensive worldwide cooperation network, followed by China and Germany. June, C.H. published the most papers with the most citations, while Maude, S.L. ranked first among the co-cited authors. The hotspots in CAR-based therapy research were multiple myeloma, safety and toxicity, solid tumors, CAR-engineered immune cells beyond T cells, and gene editing.

**Conclusion:**

CAR-based immunotherapy is a promising treatment for cancer patients, and there is an emerging movement toward using advanced gene modification technologies to overcome therapeutic challenges, especially in solid tumors, and to generate safer and more effective universal CAR-engineered cell products.

## Introduction

Among different cancer therapy strategies, immunotherapy has attracted great attention from clinical researchers worldwide. Adoptive cell transfer (ACT) therapies represent one of the most promising treatments, among which chimeric antigen receptor (CAR)-based therapy has shown great potential. The concept of CAR means that an engineered receptor with predefined specificity is grafted onto immune cells through gene editing to endow the cells with the ability to recognize target molecules without MHC restriction (1). The CAR structure consists of 3 major parts, specifically, the extracellular region with the single-chain fragment variant (scFv) that recognizes the antigen, the fundamental hydrophobic alpha helix transmembrane domain, and the core intracellular domain that recruits and phosphorylates downstream protein molecules by conformational changes (2). CAR was first proposed by the Israeli scientist Eshhar in 1989 (3) and was soon widely studied but went through a long period before being used in clinical practice. The first generation contained only the CD3ζ intracellular domain, which led to hypoproliferation and hypocytotoxicity (4); this was improved in the 2^nd^ and 3^rd^ generations by adding one or both costimulation signals, including CD28, CD134 and CD137 (5–7). The 4^th^ generation, also known as T cells redirected for universal cytokine-mediated killing (TRUCKs), includes the expression of a desired cytokine upon CAR activation (8), while in the fifth generation, the addition of the intracellular domains of cytokine receptors such as interleukin (IL)-2 receptor β-chain (IL-2Rβ) fragments has greatly strengthened the activation and proliferation ability of CAR-T cells (9). Of note, in 2017, the FDA approved the use of two CD19-targeting CAR-T products, CTL-019 (Kymriah) from Novartis and Yescarta from Gilead, in pediatric relapsed or refractory acute lymphoblastic leukemia (AML) and adult relapsed or refractory large B cell lymphoma, respectively (10, 11), which indicated that the CAR-T era had finally arrived.

Beyond CAR-T cells, other immune effector cells with CAR modification were under study simultaneously, including natural killer (NK) cells, invariant NK T (iNKT) cells, γδ T cells, macrophages and dendritic cells (DCs). These cell types display different characteristics that outperform T cells in particular aspects. For example, the risk of graft versus host disease (GVHD) mediated by the T-cell receptor can be completely avoided when NK-cells, iNKT or γδ T cells are applied (12). Moreover, multiple killing mechanisms beyond perforin and granzyme, the modulation of antitumor immunity through secretion of cytokines or direct penetration of solid tumors in response to tumor-derived chemokines make it possible to obtain better curative effects in these cell platforms for CAR-based immunotherapies (13). In fact, dozens of clinical trials of different CAR-based immune effector cells are under evaluation (12, 14).

Although tremendous efforts have been invested in CAR-modified cell therapy in cancers, the most promising clinical results have come mostly from hematopoietic malignancies and seldom from solid tumors (15). Research teams from all over the world have been exploring the reasons and potential solutions, but the most feasible ways out of this dilemma remain unclear. To better answer this question, it is necessary to understand the majority of the research foundation and build up a multidimensional research network through longitudinal and global perspectives to illustrate future directions. By bibliometric analysis, a comprehensive and effective scientific information analysis method, we can quantitatively evaluate the contributions of authors, institutions and countries and the links among them (16). More importantly, such analysis could provide a valuable basis for defining the frontiers of and trends in the research field (17).

To date, only a few scientometric studies that focused on CAR-T cells in CAR-based therapy have been presented. Here, we performed a more comprehensive bibliometric analysis of CAR-based immune cell therapy based on sufficient literature and updated data through different analysis methods, aiming to draw a global research network map and determine the next pivotal frontiers.

## Materials and Methods

### Data Source and Search Strategies

A literature search was performed using the Web of Science Core Collection (WoSCC) database on October 1^st^, 2021. To conduct a thorough search, we used the following strategy: TS = (tumo$r* OR cancer* OR carcinoma* OR sarcoma* OR neoplas* OR malignanc*) AND TS=((chimeric antigen receptor*) OR (chimeric T cell receptor*) OR “CAR-T” OR “CAR-T cell” OR “CAR therapy” OR (chimeric NK cell receptor*) OR “CAR-NK” OR “CAR-iNKT” OR “CAR-γδT” OR “CAR-macrophage” OR “CAR-M” OR Kymriah OR Yescarta). Only articles and reviews written in English and published from 2001 to 2021 were eligible to be included. Two researchers (ZO and LQ) manually screened the titles, abstracts and full texts to exclude irrelevant literature and discussed any potential disagreements. Finally, 5981 documents were included. The screening strategy is shown in **Supplementary Figure 1**.

### Data Extraction and Bibliometric Analysis

Full records and cited references of all the documents in WoSCC were collected and downloaded in txt or BibTeX format and then imported to CiteSpace 5.8R1, 64 bits (Drexel University, Philadelphia, PA, USA), Microsoft Excel 2019, VOSviewer 1.6.17 (Leiden University, The Netherlands) or R (Version 4.0.2), according to the software required for data analysis and visual analysis.

Microsoft Office Excel was used to analyze the trends of annual publications and citations of the included literature. The Bibliometrix and Biblioshiny packages in R were used to conduct collaboration network analysis among countries. VOSviewer software was used to analyze country or organization distributions, author contributions, core journals, keyword co-occurrences with coauthorship, co-citation, and co-occurrence analyses in default settings and displayed visualizations of cooperative networks of these items. We also performed the co-citation analysis in co-cited references and the combination of co-cited references and keywords by CiteSpace software, with the following settings: The time slices were from January 2001 to December 2020, with 1 year per slice. In each slice, a modified g-index $\left(g2\;\leq \;k\;Si\;\leq_{g\;i}^{c},\;k\in Z+,\;k\;=\;25\right)$ was set. The maintenance threshold for burst detection in authors and references was set to 4 years. Other parameters were set to the default settings.

### Research Ethics

We conducted the study using scientometric data with no *in vivo* data from animal or human subjects. Therefore, permission from the ethics committee was not necessary in this study.

## Results

### Overview and Analysis of the Publication and Citation Trends

A total of 7255 studies were identified after a thorough search of the WoSCC database. Document types other than articles and reviews, non-English papers and irrelevant studies were excluded (**Supplementary Figure 1**). No duplicated studies were found. Finally, 5981 studies were included in the bibliometric analysis. The total number of citations for the retrieved articles was 224,968, and the mean citations per article was 37.61. The H-index of all the selected publications was 197.

**Figure 1:**
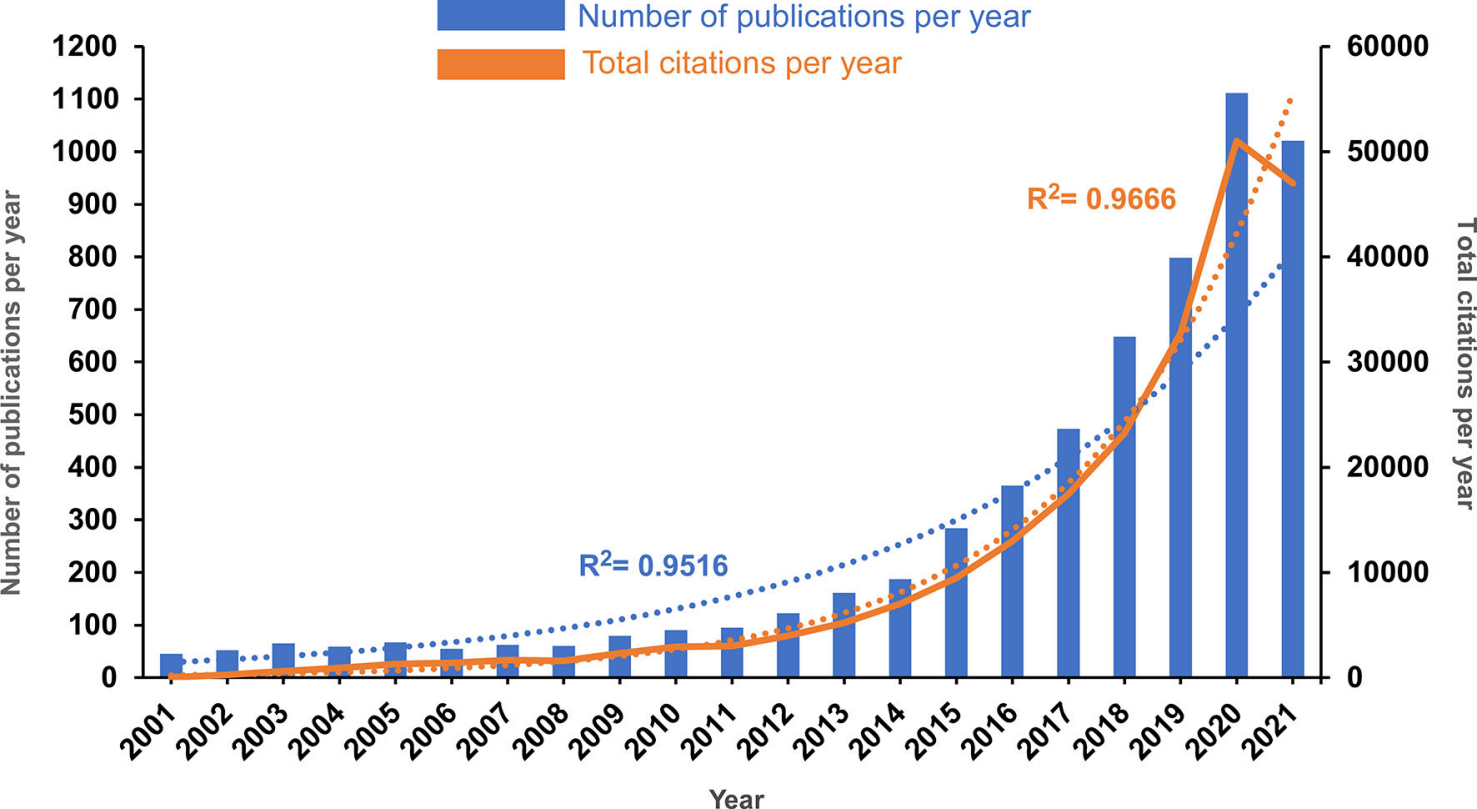
The annual publication and annual citation trends in the past 20 years. The blue bars represent the number of publications per year, and the orange solid curve represents the total number of citations per year. The blue and orange dotted lines represent the trend-fitted curves using exponential functions. The correlation coefficients (R^2^) are displayed in the figure.

As shown in **Figure 1**, the annual number of publications concerning CAR-based immunotherapy slowly increased from 45 in 2001 to 95 in 2011. From 2012 to 2020, the number of publications per year grew rapidly, reaching 1111 in 2020. The number of publications in 2021 was slightly lower than that in 2020, perhaps because the search included only three quarters of 2021. However, since the correlation between number of publications and publication year was significant (correlation coefficient R^2 =^ 0.9516), it is convincing that publication this year will establish a new maximum. Correspondingly, the number of annual citations exhibited a similar upward trend, steadily increasing from 40 in 2001 to 2999 in 2011, exploding from 3987 in 2012 to 50991 in 2020, and exhibiting a small decline to 46976 in 2021, with an even stronger correlation (correlation coefficient R^2 =^ 0.9666).

Overall, the publication and citation number results indicated that research on CAR-based immunotherapy in cancer is still in a rapid growth phase, provoking great attention for future research.

### Analysis of Productive Journals

**Figure 2:**
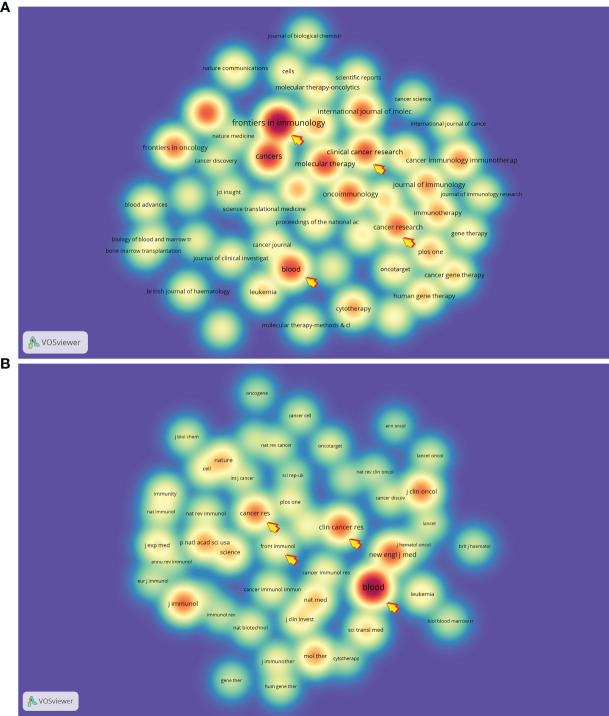
The spectral density map of **(A)** citing and **(B)** co-cited journals. The arrows point to the journals that are present in both panels.

We found that a total of 5981 papers concerning CAR-based immunotherapy in cancer were produced by 1030 journals. Among these publication sources, the journal *Frontiers in Immunology* ranked first in production (252, 4.21%), followed by *Cancers* (155, 2.59%), *Molecular Therapy* (130, 2.17%) and *Blood* (127, 2.12%). Of these, the highest impact factor (IF, 2020) among the top 10 most productive journals was earned by *Blood* (22.113), followed by *Journal for Immunotherapy of Cancer* (13.751), *Cancer Research* (12.701) and *Clinical Cancer Research* (12.531, **Table 1**). Half of the top 10 citing journals belonged to Q1 in journal citations reports (JCR), and 70% were from the US (**Table 1**). Furthermore, as listed in **Table 1**, all of top 10 co-cited journals were cited over 8000 times, accounting for more than 30% of the total citations, with *Blood* (37,798, 8.15%) again being the highest, and *New England Journal of Medicine* (16,573, 3.57%) and *Clinical Cancer Research* (15,154, 3.26%) ranking second and third. Ninety percent of these co-cited journals had IF values over 10 and belonged to Q1 in JCR. We also visualized the top 50 citing and co-cited journals with a spectral density map (**Figure 2**) and found that some of the journals co-occurred in both maps, for example, *Blood*, *Frontiers in Immunology*, *Clinical Cancer Research* and *Cancer Research*, indicating that these journals have maintained close contact with the CAR-based immunotherapy research field.

**Table 1:** Top 10 journals and co-cited journals related to CAR-based immunotherapy.

Rank	Journal	Counts (%)	IF (2021)	JCR	Country	Rank	Co-cited Journal	Counts (%)	IF (2021)	JCR	Country
1	Frontiers in Immunology	252(4.21)	7.561	Q2	Switzerland	1	Blood	37798 (8.15)	22.113	Q1	US
2	Cancers	155(2.59)	6.639	Q2	Switzerland	2	New England Journal of Medicine	16573 (3.57)	91.245	Q1	US
3	Molecular Therapy	130(2.17)	11.454	Q1	US	3	Clinical Cancer Research	15154 (3.26)	12.531	Q1	US
4	Blood	127(2.12)	22.113	Q1	US	4	Journal of Immunology	13870 (2.99)	5.422	Q2	US
5	Clinical Cancer Research	111(1.85)	12.531	Q1	US	5	Cancer Research	13614 (2.93)	12.701	Q1	US
6	Journal for Immunotherapy of Cancer	111(1.85)	13.751	Q3	UK	6	Journal of Clinical Oncology	13454 (2.90)	44.544	Q1	US
7	International Journal of Molecular Sciences	99(1.65)	5.923	Q2	US	7	Molecular Therapy	10809 (2.33)	11.454	Q1	US
8	Oncoimmunology	98(1.63)	8.110	Q1	US	8	Proceedings of the National Academy of Sciences of the United States of America	10597 (2.28)	11.205	Q1	US
9	Cancer Research	96(1.60)	12.701	Q1	US	9	Science	8492 (1.83)	47.728	Q1	US
10	Frontiers In Oncology	93(1.55)	6.244	Q2	US	10	Nature Medicine	8465 (1.82)	53.440	Q1	US

IF, impact factor; JCR, journal citation reports; US, The United States; UK, The United Kingdom.

### Analysis of Active Countries and Organizations

The literature included for analysis was produced by 83 countries over the last two decades (articles coauthored by individuals from more than one country or organization were counted multiple times by VOSviewer). Among these countries, the United States published the most papers (n=3088), accounting for 38.82% of the included studies and the most citations (163148), which outnumbered the total citations from the 2^nd^ to 10^th^ countries (**Table 2**). The next most productive countries included China (n=1099, 13.82%), Germany (n=661, 8.31%) and the United Kingdom (n=366, 4.6%), and all top 10 countries exceeded the average number of publications (n=95.83). Interestingly, China ranked 2^nd^ in publications but the last in average document citations (n=17.68), while Australia and Canada displayed the opposite pattern, ranking 2^nd^ and 3^rd^ in citation per paper (n=49.41 and 48.30, respectively) with fewer publications (n=177, 2.23% and 160, 2.01%, respectively). These may be related to access to the literature and the restriction of language selected.

**Table 2:** Top 10 countries and organizations related to CAR-based immunotherapy.

Rank	Country	Counts (%)	Total citations	Citations per article	Rank	Organizations	Counts (%)	Total citations	Citations per article
1	US	3088(38.82)	163148	52.83	1	University of Pennsylvania (US)	344 (2.12)	35010	101.77
2	China	1099(13.82)	19425	17.68	2	Memorial Sloan Kettering Cancer Center (US)	243 (1.50)	21389	88.02
3	Germany	661(8.31)	26204	39.64	3	National Cancer Institute (US)	204 (1.26)	24261	118.93
4	UK	366(4.60)	12985	35.48	4	The University of Texas MD Anderson Cancer Center (US)	197 (1.21)	13305	67.54
5	Italy	308(3.87)	10609	34.44	5	Baylor College of Medicine (US)	160 (0.99)	13651	85.32
6	Japan	228(2.87)	7516	32.96	6	University of Washington (US)	160 (0.99)	10614	66.34
7	France	211(2.65)	6626	31.40	7	Harvard University (US)	152 (0.94)	3861	25.40
8	Australia	177(2.23)	8746	49.41	8	Fred Hutchinson Cancer Research Center (US)	131 (0.81)	10604	80.95
9	Canada	160(2.01)	7728	48.30	9	Texas Children’s Hospital (US)	111 (0.68)	10173	91.65
10	Netherlands	145(1.82)	5689	39.23	10	Stanford University (US)	100 (0.62)	4349	43.49

US, The United States; UK, The United Kingdom.

**Figure 3:**
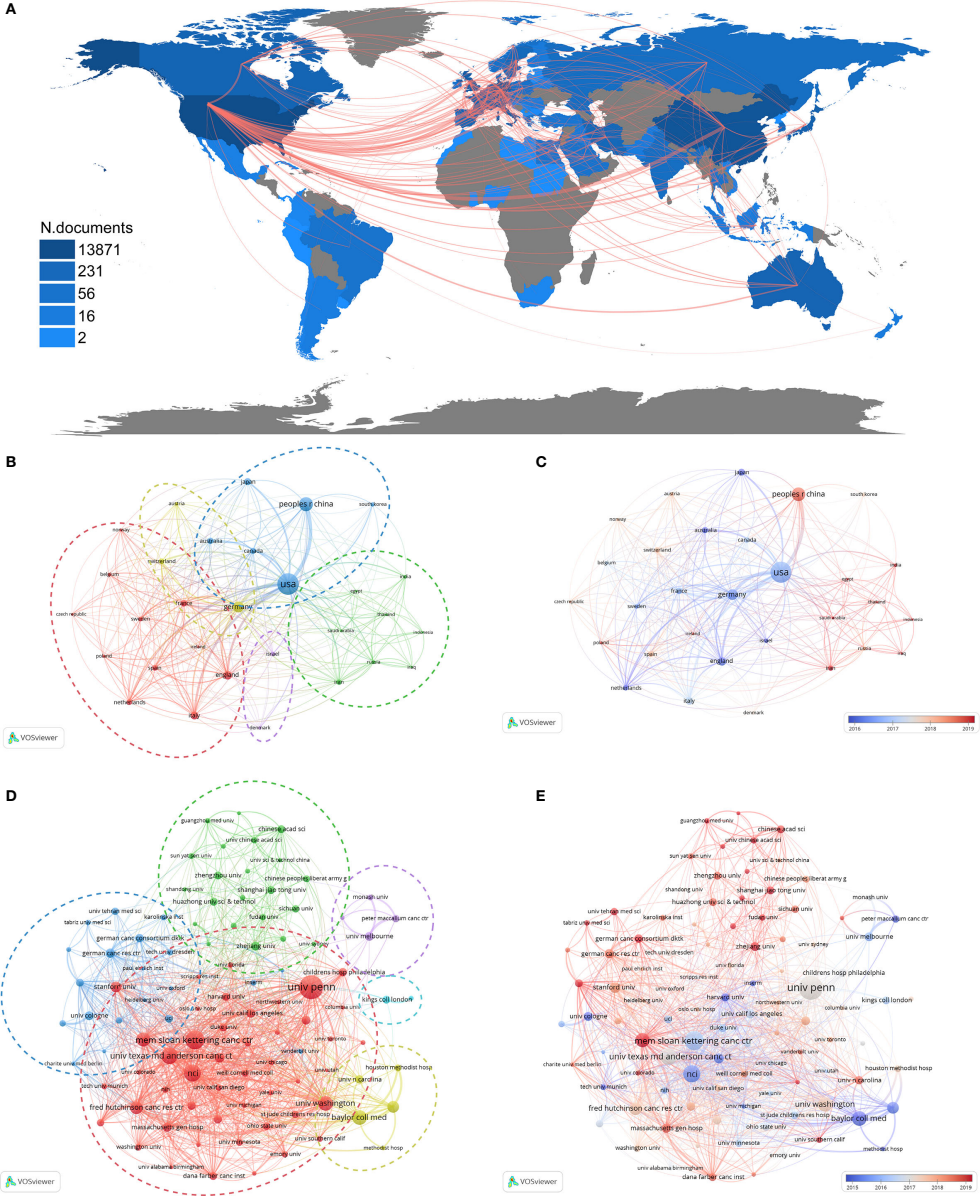
Collaboration networks among countries and among institutions. **(A)** The collaboration map among countries. Color shades represent the number of publications in each country, and red lines indicate cooperation between two countries. Each node in **(B–E)** represents a country or an institution, and each line represents a link between two countries or institutions. The dotted circles in different colors in **(B, D)** indicate the corresponding clusters of countries and institutions. **(B)** The cooperation network among the top 30 most productive countries. **(C)** The time-overlay map of the cooperation network among the top 30 most productive countries. **(D)** The cooperation network among the top 100 most productive institutions. **(E)** The time-overlay map of the cooperation network among the top 100 most productive institutions.

To explore the collaborations between countries, we used Bibliometrix and Biblioshiny packages and VOSviewer to analyze the data and generate visual outputs. As shown in **Figure 3A**, the collaboration network among countries was complicated and extensive. The cooperation centers, displayed by the junctions of red lines, are mainly located in North America (US), Asia (China), Europe (Germany) and Oceania (Australia). The country coauthorship network of the top 30 countries was automictically clustered into 5 categories, as indicated by 5 colors (**Figure 3B**). The time-overlay visualization map showed that the production of these countries has been concentrated within the last 5 years (**Figure 3C**). Considering these observations together with the clustering results, it was supposed that the green cluster (mostly Asian countries) was more newly active than other clusters, while in each cluster, some of the members have become more active in the last 3 years, such as China in the blue cluster (**Figures 3B, C**).

A total of 4557 organizations produced at least 1 paper related to CAR-based immunotherapy in cancer. The top 10 most productive institutions are shown in **Table 2**. All of these institutions are located in the US and have published over 100 papers in the past 20 years, with the number of publications ranging from 100 (Stanford University) to 344 (University of Pennsylvania), total citations ranging from 3861 (Harvard University) to 35010 (University of Pennsylvania), and citations per paper ranging from 25.40 (Harvard University) to 118.93 (National Cancer Institute).

The cooperation of the top 100 research institutions was likewise analyzed by a coauthorship network. Six clusters were displayed (**Figure 3D**), mainly indicating regional collaboration. The largest cluster (red) consists mainly of institutions in the US, the blue cluster in Germany, the green cluster in China, the purple cluster in Australia, the light blue cluster in the UK and the yellow cluster for institutions in Houston, Texas (US). Among these clusters, the red, yellow and purple clusters were most active from approximately 2015 to 2017, while the green and blue clusters were more active in the last 3 years, indicating that institutions in the US, UK and Australia began CAR-T research earlier, but those in China and Germany, such as Zhejiang University, Shanghai Jiao Tong University, Sun Yat-Sen University, and the German Cancer Consortium (DKTK), were close behind and have produced more newer studies (**Figure 3E**).

### Analysis of Authors and Coauthorship

**Figure 4:**
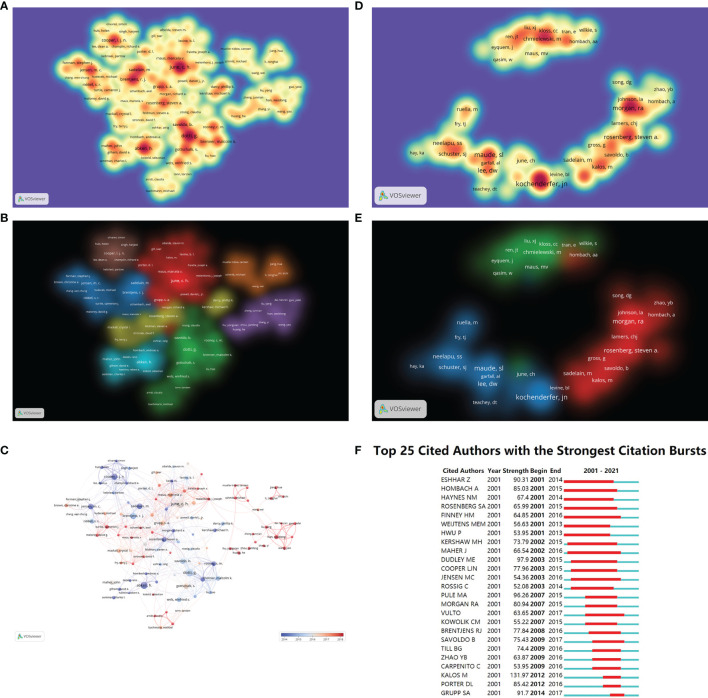
Scholar cooperation maps. **(A)** The spectral density map of the top 100 most productive authors. **(B)** The cluster-overlay map of the top 100 most productive authors. **(C)** Time-overlay maps of the top 100 most productive authors. Each node represents an author, and each line represents the link between two authors. **(D)** The spectral density maps of the top 100 most co-cited authors. **(E)** The cluster-overlay map of the top 100 most co-cited authors. **(F)** The top 25 cited authors with the strongest citation bursts.

A total of 26367 authors participated in publishing the literature involved in this analysis, and 22.2% of these authors have published more than one paper. The top 100 most productive authors and co-cited authors are displayed in spectral density maps in **Figures 4A, D**, while the details of the top 20 authors in both rankings are listed in **Table 3**. Among the top 20 most productive authors, 36 to 104 papers per person were authored, receiving 1589 to 22985 citations, while the total citations of the co-cited authors ranged from 899 to 2868. Notably, June, C.H., one of the pioneers of 4-1BB-CAR-T cells (18), had the most published papers (104) with the most citations (22985) and ranked the 17^th^ among co-cited authors. In addition, 4 other authors, including Brentjens, R.J., Sadelain, M., Grupp, S.A. and Rosenberg, S.A., were also within the top 20 in both rankings, demonstrating their academic authority in this research field. The cooperation networks among authors and among co-cited authors were grouped into 7 and 3 clusters according to the closeness of their connections (**Figures 4B, E**), respectively. When combined with the overlay visualization maps of year (**Figure 4C**), it reflected that the brown and light blue clusters were most active in approximately 2014, and the blue, red, yellow and green clusters expanded over time, while the orange and purple clusters mainly published literature in the past 3 years but lack strong connections with other clusters. Among the co-cited authors, Maude, S.L. ranked first, but the centralities of all co-cited authors were below 0.1 (0-0.04), indicating that multiple researchers began to publish within a short period; thus, no single author dominates the researching field. This was also verified by the burst of co-cited authors because these researchers shared the same burst period or overlapped with each other (**Figure 4F**). The burstiness strength ranged from 52.08 (Rossig, C.) to 131.97 (Kalos, M.) among the top 25 co-cited authors with the strongest bursts (**Figure 4F**) but was inconsistent with the top 20 co-cited authors in **Table 3** because several authors did not reach the threshold of 4 years of burst maintenance.

**Table 3:** Top 20 most relevant authors and co-cited authors related to CAR-based immunotherapy.

Rank	Author	Counts	Total citations	Citations per article	Rank	Co-cited author	Total citations	Centrality	Burstiness strength
1	June, Carl H	104	22985	221.01	1	Maude, Shannon L	2868	0.04	–
2	Dotti, Gianpietro	98	9797	99.97	2	Kochenderfer, James N	2848	0.02	50.56
3	Abken, Hinrich	80	4126	51.58	3	Brentjens, Renier J	2118	0.04	77.84
4	Brentjens, Renier J	68	9711	142.81	4	Lee, Daniel W	1886	0.02	–
5	Cooper, Laurence JN	64	4216	65.88	5	Porter, David L	1855	0.02	85.42
6	Savoldo, Barbara	60	7139	118.98	6	Rosenberg, Steven A	1807	0.03	65.99
7	Sadelain, Michel	59	12791	216.80	7	Morgan, Richard A	1787	0.01	80.94
8	Jensen, Michael C	58	7525	129.74	8	Neelapu, Sattva S	1441	0.01	–
9	Gottschalk, Stephen	53	3164	59.70	9	Turtle, Cameron J	1349	0.02	–
10	Brenner, Malcolm K	49	6685	136.43	10	Sadelain, Michel	1226	0.01	32.14
11	Grupp, Stephan A	49	9528	194.45	11	Grupp, Stephan A	1213	0.01	91.7
12	Rosenberg, Steven A	48	13476	280.75	12	Davila, Marco L	1127	0.01	43.95
13	Riddell, Stanley R	46	7788	169.30	13	Schuster, Stephen J	1064	0.03	–
14	Rooney, Cliona M	46	6835	148.59	14	Kalos, Michael	999	0.00	131.97
15	Maus, Marcela V	43	4328	100.65	15	Brudno, Jennifer N	996	0.01	–
16	Heslop, Helen E	42	6617	157.55	16	Chmielewski, Markus	960	0.01	–
17	Han, Weidong	42	2055	48.93	17	June, Carl H	945	0.01	–
18	Maher, John	41	1589	38.76	18	Kershaw, Michael H	907	0.01	73.79
19	Wels, Winfried S	41	3061	74.66	19	Gattinoni, Luca	905	0.01	42.02
20	Forman, Stephen J	40	3850	96.25	20	Brown, Christine E	899	0.02	–

-, the burstiness strength was missing because the threshold of maintenance of co-cited authors was set to 3 years.

### Analysis of the Research Field From Co-Cited References and Keywords

**Figure 5:**
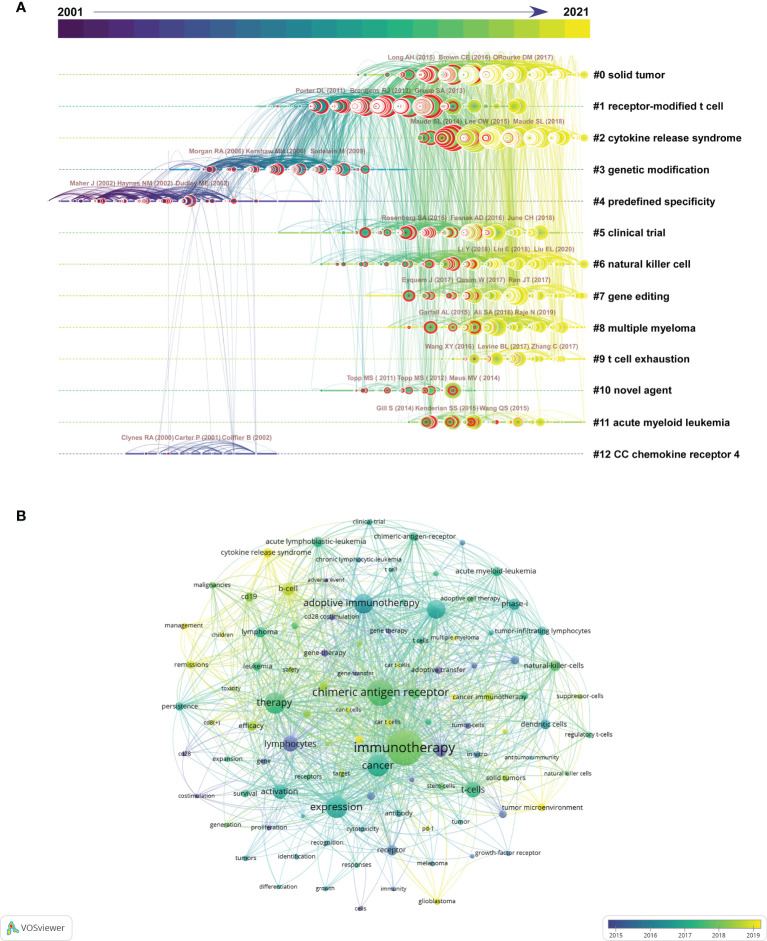
Knowledge map of co-cited references. **(A)** Timeline view of 13 clusters of co-cited references and the corresponding tags. Each node represents an article or review, and the red circle around the node represents a reference burst. Each line represents a link between two studies. The top 3 co-cited references of each cluster are also displayed above the nodes. **(B)** The time-overlay network map of the top 100 co-occurring keywords.

To explore where researchers have been and where they are going in CAR-based immunotherapy, we analyzed the co-citation network of references and keywords by CiteSpace and VOSviewer. First, among 5981 original documents, 1873 cited references from 2665 citing papers were selected automatically by CiteSpace to form the co-citation network (**Figure 5A** and **Supplementary Figure 2**). The top 20 co-cited references were all published in internationally renowned journals (IF>14.808, Q1), with over 344 citations (**Table 4**). Most of these papers were clinical studies, with only one preclinical study and one review. The top 10 co-cited references with updated citation data related to hematopoietic malignances and solid tumors were also displayed in **Tables S1**, **S2**, respectively. In addition, the top 25 references with the strongest citation bursts showed that the majority were frequently cited in the last decade, suggesting that the research field is still progressing (**Supplementary Figure 3**). The included references were clustered into 13 groups based on their major research topic (**Figure 5A** and **Supplementary Figure 2**). Cluster #4, predefined specificity (mean year = 2000), #12, CC chemokine receptor 4 (mean year = 2002) and #3, genetic modification (mean year = 2006) appeared initially, followed by #1, receptor-modified T cell (mean year = 2011) and #10, novel agent (mean year = 2012) and #5 clinical trial (mean year = 2014), while #6, natural killer cell (mean year = 2015), #7, gene editing (mean year = 2015), #11, acute myeloid leukemia (mean year = 2015), #0, solid tumor (mean year = 2016), #2, cytokine release syndrome (mean year = 2017), #8, multiple myeloma (mean year = 2017) and #9, T cell exhaustion (mean year = 2017) were new focuses of research. The top 3 most cited references of each cluster are also displayed by the 3 largest circles in each group (**Figure 5A**).

**Table 4:** Top 20 co-cited references related to CAR-based immunotherapy.

Rank	Reference	Citations	Author	Year	Type	Journal	IF	JCR
1	Chimeric antigen receptor t cells for sustained remissions in leukemia	958	Maude Shannon L	2014	Article(CT)	The New England Journal of Medicine	91.245	Q1
2	T cells expressing CD19 chimeric antigen receptors for acute lymphoblastic leukaemia in children and young adults: a phase 1 dose-escalation trial	813	Lee Daniel W	2015	Article(CT)	LANCET	79.321	Q1
3	Tisagenlecleucel in children and young adults with B-cell lymphoblastic leukemia	799	Maude Shannon L	2018	Article(CT)	The New England Journal of Medicine	91.245	Q1
4	Axicabtagene ciloleucel CAR T-cell therapy in refractory large B-cell lymphoma	766	Neelapu Sattva S	2017	Article(CT)	The New England Journal of Medicine	91.245	Q1
5	Chimeric Antigen Receptor–Modified T Cellsfor Acute Lymphoid Leukemia	662	Grupp Stephan A	2013	Article(CT)	The New England Journal of Medicine	91.245	Q1
6	Efficacy and Toxicity Management of 19-28z CAR T Cell Therapy in B Cell Acute Lymphoblastic Leukemia	637	Davila Marco L	2014	Article(CT)	Science Translational Medicine	17.956	Q1
7	CD19 CAR–T cells of defined CD4+:CD8+ composition in adult B cell ALL patients	579	Turtle Cameron J	2016	Article(CT)	Journal of Clinical Investigation	14.808	Q1
8	Chemotherapy-Refractory Diffuse Large B-Cell Lymphoma and Indolent B-Cell Malignancies Can Be Effectively Treated with Autologous T Cells Expressing an Anti-CD19 Chimeric Antigen Receptor	559	Kochenderfer James N	2015	Article(CT)	Journal of Clinical Oncology	44.544	Q1
9	CD19-targeted T cells rapidly induce molecular remissions in adults with chemotherapy-refractory acute lymphoblastic leukemia	539	Brentjens Renier J	2013	Article(CT)	Science Translational Medicine	17.956	Q1
10	Chimeric antigen receptor T cells persist and induce sustained remissions in relapsed refractory chronic lymphocytic leukemia	497	Porter David L	2015	Article(CT)	Science Translational Medicine	17.956	Q1
11	Chimeric Antigen Receptor–Modified T Cells in Chronic Lymphoid Leukemia	475	Porter David L	2011	Article(CR)	The New England Journal of Medicine	91.245	Q1
12	Long-Term Follow-up of CD19 CAR Therapy in Acute Lymphoblastic Leukemia	469	Park Jae H	2018	Article(CT)	The New England Journal of Medicine	91.245	Q1
13	Regression of Glioblastoma after Chimeric Antigen Receptor T-Cell Therapy	398	Brown Christine E	2016	Article(CR)	The New England Journal of Medicine	91.245	Q1
14	Chimeric antigen receptor T−cell therapy — assessment and management of toxicities	398	Neelapu Sattva S	2017	Review	Nature Reviews Clinical Oncology	66.675	Q1
15	Tisagenlecleucel in Adult Relapsed or Refractory Diffuse Large B-Cell Lymphoma	393	Schuster Stephen J	2019	Article(CT)	The New England Journal of Medicine	91.245	Q1
16	B-cell depletion and remissions of malignancy along with cytokine-associated toxicity in a clinical trial of anti-CD19 chimeric-antigen-receptor–transduced T cells	390	Kochenderfer James N	2012	Article(CT)	Blood	22.113	Q1
17	T Cells with Chimeric Antigen Receptors Have Potent Antitumor Effects and Can Establish Memory in Patients with Advanced Leukemia	387	Kalos Michael	2011	Article(CT)	Science Translational Medicine	17.956	Q1
18	Chimeric Antigen Receptor T Cells in Refractory B-Cell Lymphomas	371	Schuster Stephen J	2017	Article(CT)	The New England Journal of Medicine	91.245	Q1
19	4-1BB costimulation ameliorates T cell exhaustion induced by tonic signaling of chimeric antigen receptors	362	Long Adrienne H	2015	Article	Nature Medicine	53.440	Q1
20	A single dose of peripherally infused EGFRvIII-directed CAR T cells mediates antigen loss and induces adaptive resistance in patients with recurrent glioblastoma	344	O’Rourke Donald M	2017	Article(CT)	Science Translational Medicine	17.956	Q1

CT, clinical trial; CR, case report; IF, impact factors; JCR, journal citation reports.

Keywords are also essential in hotspot analysis. The top 100 keywords with the highest co-occurrence were calculated by VOSviewer and are displayed in **Figure 5B**, showing a comprehensive but clear network. The detailed data of the top 25 co-occurrence keywords are listed in **Table 5**. Among them, the basic concepts “chimeric antigen receptor”, “immunotherapy” and “cancer” ranked in the top 3 (**Table 5**), while “cytokine release syndrome”, “tumor microenvironment”, “multiple myeloma” and “glioblastoma” seemed to represent the forefront of the research field (**Figure 5B**). Moreover, the top 25 keywords with the strongest citation bursts are listed in **Table 5**, showing that most of the keywords were derived 10 years ago, and some of them still have high occurrences, such as “*in vivo*” (2002-2017), “antitumor activity” (2010-2017), and “adoptive immunotherapy” (2011-2016). The target antigens used in the published CAR-based immunotherapies in both liquid cancers and solid tumors were summarized in **Table S3**. Co-occurrence analysis was applied to reveal the popularity of these targets.

**Table 5:** Top 25 co-occurrence keywords and top 25 keywords with strongest citations burst related to CAR-based immunotherapy.

Rank	Keyword	Occurrences	Total link strength	Rank	Keyword with strongest citations burst	Strength	Begin year	End year
1	chimeric antigen receptor	2005	24140	1	chimeric receptor	41.93	2001	2013
2	immunotherapy	1894	22855	2	monoclonal antibody	40.14	2001	2012
3	cancer	949	11358	3	lymphocyte	38.51	2001	2013
4	expression	946	10455	4	single chain	17.7	2001	2013
5	therapy	919	10055	5	tumor cell	17.56	2001	2011
6	adoptive immunotherapy	820	9905	6	signal transduction	16.04	2001	2009
7	antitumor-activity	765	9259	7	cell	15.89	2001	2008
8	CAR-T cells	719	8938	8	receptor	15.41	2001	2010
9	natural killer cells	607	7642	9	adoptive transfer	15.39	2001	2015
10	t-cells	599	7256	10	*in vivo*	30.17	2002	2017
11	lymphocytes	578	6375	11	tumor necrosis factor	19.25	2002	2010
12	activation	537	6020	12	cd28 costimulation	20.96	2003	2015
13	phase-i trial	490	6308	13	proliferation	17.7	2003	2013
14	adoptive cell therapy	467	5897	14	cancer regression	18.72	2007	2015
15	b cell	430	5047	15	*in vivo* persistence	16	2007	2016
16	acute lymphoblastic leukemia	418	5093	16	gene therapy	35.2	2009	2015
17	*in-vivo*	379	4434	17	metastatic melanoma	18.49	2009	2015
18	cytokine release syndrome	338	3907	18	cd28	18.25	2010	2015
19	receptor	337	3779	19	antitumor activity	15.92	2010	2017
20	tumor infiltrating lymphocytes	334	4325	20	adoptive immunotherapy	38.6	2011	2016
21	lymphoma	334	3889	21	adverse event	18.28	2011	2014
22	survival	333	3877	22	persistence	20.26	2012	2016
23	dendritic cells	322	4243	23	clinical trial	15.8	2013	2016
24	tumor microenvironment	318	4081	24	modified t cell	19.27	2015	2018
25	gene therapy	318	4042	25	CAR-T	22.13	2019	2021

**Figure 6:**
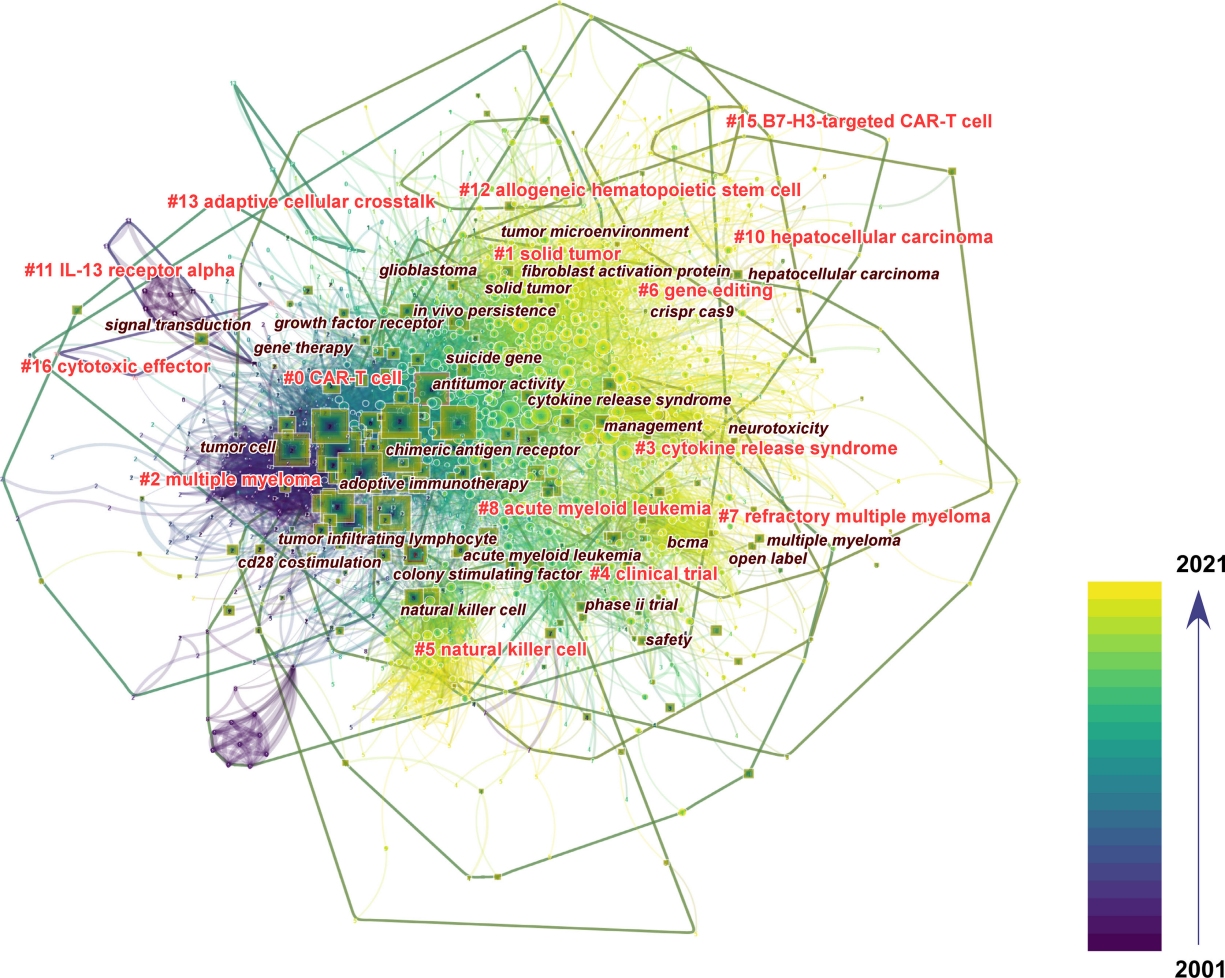
The mixed visualization map of both co-cited references and keywords. Each node represents an article or review, each square represents a keyword, and each frame represents a cluster. The size of each node and square represent the numbers of co-citations and co-occurrences, respectively. The labels of the clusters and the most relevant keywords were also displayed adjacent to the frames. The color indicates the publication year of the articles.

Unfortunately, these keywords are general and only partially relate to the frontiers of CAR-based immunotherapy. Therefore, to sufficiently unveil the hotspots, we performed mixed scientometric analysis with co-cited references and keywords and displayed the result with an overlay network map (**Figure 6**). Different from the former 13 clusters, 14 out of 16 clusters are shown in this section (2 irrelevant clusters were hidden), together with the most correlative keywords. The keywords were mainly concentrated in earlier times, especially for cluster #0 labelled “CAR-T cells” and #2 labelled “multiple myeloma”, but the new keywords with fewer occurrences should not be neglected. Generally, the clusters could be mainly divided into the following parts: the basic concept of CAR (including cluster #0 and #16, and the related keywords “chimeric antigen receptor”, “adoptive immunotherapy”, “antitumor activity”, etc.), solid tumor (including #1, #6, #10, #11, #13 and #15, and keywords “solid tumor”, “glioblastoma”, “tumor microenvironment”, “fibroblast activation protein”, etc.), hematopoietic malignancies (including #2, #7, #8 and #12, and keywords “acute myeloid leukemia”, “multiple myeloma”, “bcma”, etc.), safety (#3 and keywords “cytokine release syndrome”, “neurotoxicity”, “management”, etc.), clinical trials (including #4 and keywords “phase ii trial”, “open label”, etc.), CAR-based innate cell therapy (including #5 and keywords “natural killer cells”, “colony stimulating factor”, etc.) and genetic modification (#6 and keywords “CRISPR”, “Cas9”, “suicide gene”, etc.). Furthermore, the most recent clusters, including #1 (mean year = 2016), #6 (mean year = 2017), #10 (mean year = 2017) and #15 (mean year = 2018), mainly focus on the treatment of solid tumors. We then took a closer look at cluster #15, which represented the leading edge of CAR-based immunotherapy in solid tumors. There were 5 cited references in #15 with between 11 and 57 citations, mainly related to B7-H3-targeted CAR-T cells in brain tumors (**Table 6**). The top 5 citing references that cited the 5 cited references mentioned above are also listed in **Table 7** and were equally important because they cover the same topics, indicating that these studies were closely related to the frontier of this research.

**Table 6:** Cited References in cluster 15 of the overlay analysis of co-cited references and keywords.

Rank	Reference	Citations	Author	Year	Type	Journal	IF	JCR
1	CAR T cells targeting B7-H3, a Pan-Cancer Antigen, Demonstrate Potent Preclinical Activity Against Pediatric Solid Tumors and Brain Tumors	57	Majzner Robbie G.	2019	Article	Clinical Cancer Research	12.531	Q1
2	Antitumor Responses in the Absence of Toxicity in Solid Tumors by Targeting B7-H3 *via* Chimeric Antigen Receptor T Cells	46	Du Hongwei	2019	Article	Cancer Cell	31.743	Q1
3	B7-H3 as a Novel CAR-T Therapeutic Target for Glioblastoma	17	Tang Xin	2019	Article	Molecular Therapy: Oncolytics	7.200	Q4
4	B7-H3-redirected chimeric antigen receptor T cells target glioblastoma and neurospheres	12	Nehama Dean	2019	Article	EBioMedicine	8.143	Q2
5	Eradication of Tumors through Simultaneous Ablation of CD276/B7-H3-Positive Tumor Cells and Tumor Vasculature	11	Seaman Steven	2017	Article	Cancer Cell	31.743	Q1

IF, impact factors; JCR, journal citation reports.

**Table 7:** Top 5 citing References in cluster #15 of the overlay analysis of co-cited references and keywords.

Coverage	Reference	Citations	Author	Year	Type	Journal	IF	JCR
4	B7-H3-targeted CAR-T cells exhibit potent antitumor effects on hematologic and solid tumors	18	Zhang Zongliang	2020	Article	Molecular Therapy: Oncolytics	7.200	Q4
4	Targeting B7-H3 immune checkpoint with chimeric antigen receptor-engineered natural killer cells exhibits potent cytotoxicity against non-small cell lung cancer	10	Yang Shuo	2020	Article	Frontiers in Pharmacology	5.810	Q1
4	Route of 41BB/41BBL costimulation determines effector function of B7-H3-CAR.CD28z T cells	8	Nguyen Phuong	2020	Article	Molecular Therapy: Oncolytics	7.200	Q4
4	MEK inhibitor augments antitumor activity of B7-H3-redirected bispecific antibody	5	Li Hongjian	2020	Article	Frontiers in Oncology	6.244	Q2
4	Chimeric antigen receptor T-cell therapy in glioblastoma: current and future	3	Li Long	2020	Review	Frontiers in Immunology	7.561	Q2

IF, impact factors; JCR, journal citation reports.

Taken together, these findings provide a better view of the development and front lines of research on CAR-based immunotherapy in cancers.

## Discussion

The past 20 years have witnessed the initiation and major expansion of the research field of CAR-based immunotherapy. In the first decade, major emphasis was placed on transferring concepts and theories into clinical practice, including developing the 2^nd^ and 3^rd^ generation of CAR-T cells (19), establishing preclinical studies in different kinds of cancers (20) and receiving FDA approval for the first time for CAR-T cells targeting CD19 (10, 11); thus, relatively few studies were published in this interval. After the success of CD19 CAR-T in B-cell non-Hodgkin lymphoma (B-NHL) in 2008 (21), in refractory chronic lymphocytic leukemia (CLL) in 2011 (22) and in refractory and relapsed ALL in 2013 (23), studies of CAR-based cell therapy entered the fast lane. In 2017, the FDA approved two CD19 CAR-T products, CTL-019 (Kymriah) and Yescarta, indicating the beginning of the commercialization of CAR-based cell therapy. In addition, preclinical studies and clinical trials of CAR-modified immune effect cells have emerged rapidly (12, 24). Faced with the extreme proliferation of research information, we conducted a bibliometric analysis to define the complex collaboration networks and predict the next research hotspots.

The results in this study were based on bibliographic data from published articles and reviews. Therefore, where these studies were published are of great importance, because the core sources provide basic and important evidence for the research field and affect future directions (25). According to the results, *Frontiers in Immunology* has published the most literature related to CAR-based immunotherapy and has been active in recent years, indicating that this journal has focused substantially on this field, providing an effective publishing platform for academic communications. On the other hand, the most cited journals played key roles in linking to and informing the following research (26). Our results showed that both the top 10 most cited journals and the journals with most cited references were world-famous and of high quality, confirming the importance of CAR-based immunotherapy in cancers in the future. Of note, some journals ranked first on both lists, indicating that they might act as leaders and advocates to advance the research on this topic. *Blood*, the most popular co-cited journal and the journal with the highest IF among the top 10 most productive sources, was the pioneer publishing original research on CAR-T cell therapy in hematopoietic malignancy science at the very beginning of this field (27) and continued to publish novel research on CAR-based immunotherapy (28).

Based on our analysis of cooperation among countries, institutions and authors, we may identify some trends in CAR-based immunotherapy in cancers. First, the number of countries involved in this field of research on a global scale has been growing. The collaboration networks of countries and organizations illustrated that CAR-based cell therapy has attracted attention from researchers worldwide. However, most of the published works have originated from a few countries and organizations. In the past 2 decades, production from the top 10 countries accounted for 81% of all publications, and those from the top 10 most productive institutions, which were all from the US, accounted for over 10% of all publications, implying the leadership of these countries and organizations and an imbalance of academic resources. It is noteworthy that China, the only developing country in the top 10 most productive countries, has actively taken part in this research field, especially in recent years. However, the lack of high-quality studies, as indicated by the low number of citations per study, evidently weaker connections with other countries in the collaboration maps and few top researchers with highly co-cited productions, suggested that these latecomers in such research areas should pay more attention to innovations and cooperation to find the way out.

The 2021 Dan David Prize award was presented to 3 pioneers, Rosenberg, S.A., Eshhar, Z. and June, C.H., for their great contributions to the development of CAR-T therapy. While Rosenberg established the foundation of adaptive cell transfer therapy 50 years ago (29), Eshhar empowered cytotoxic T cells (CTLs) to recognize antigens in an MHC-independent manner by gene transfer, thus becoming one of the researchers who invented the first generation of CAR-T cells (4, 30). Subsequently, Sadelain, M. and Campana, D. designed 2^nd^-generation CARs, and June was devoted to the clinical translation of CAR-T cells in cancer therapy (31). In our study, these authors were identified as the most productive or co-cited authors or authors with the strongest citation burst, which further validated the accuracy of our analytical results.

The most pressing question in this study is what the front lines of CAR-based immunotherapy in cancer are. Co-cited references (i.e., papers cited by the same study) provided the knowledge base and informed the following research (32). The top 20 co-cited references and top 25 references with the highest citation bursts were both mainly clinical case series or case reports focusing on the clinical application of CAR-T cells in ALL (33), CLL (34), lymphoma (35), and safety or toxicity management (36), indicating that the major successes of CAR-based immunotherapy have been in hematopoietic malignancies. According to the clustering results, the directions of the research field have switched from predefined specificity, CC chemokine receptor 4, genetic modification, receptor-modified T cells and clinical trials to natural killer cells, gene editing, acute myeloid leukemia, solid tumors, cytokine release syndrome, multiple myeloma and T cell exhaustion. Keyword co-occurrence can also help to identify popular research subtopics (37), but in this study, we found it insufficient to provide meaningful information. Interestingly, when we combined the keywords with co-cited references and conducted a mixed analysis, we were surprised to find 14 novel clusters, which were not exactly the same as the former clusters, that better illustrated the frontiers of the specific research field with correlated keywords. These appealing subtopics included multiple myeloma, safety and toxicity, solid tumors, CAR-engineered immune cells beyond T cells, and gene editing.

Among hematopoietic cancers, multiple myeloma (MM) has always been considered an almost incurable malignancy of plasma cells because most patients will eventually relapse or become refractory to multiple treatments (38). The emergence of CAR-T cells brought hope for patients with relapsed refractory MM (R/R MM), but conventional targets, such as CD19, may be invalid because of their infrequent expression on these cancer cells. The use of B cell maturation antigen (BCMA), a member of the tumor necrosis factor superfamily that is widely expressed on the surface of multiple myeloma cells but has limited expression on normal human cells and no expression on hematopoietic cells, might be the key breakthrough, as suggested by multiple clinical trials using BCMA-targeting CAR-T therapy (39–41). Allogeneic hematopoietic stem cell transplantation (allo-HSCT) is another hotly debated topic. The combination of allo-HSCT and CAR-T therapy seem to provide benefit for patients with advanced diseases, particularly high-risk B-cell acute lymphoblastic leukemia (B-ALL) (42). However, the ideal application sequence of the two landmark therapies, the optimal therapeutic window for post allo-HSCT CAR-T infusion, the value of CAR-T in treating peri-transplantation minimal residual disease (MRD), and the utility of CAR-base technology in treating graft-versus-host disease (GVHD), the most frequent complication after allo-HSCT, remain unclear (43).

The safety and toxicity of CAR-based therapy are ongoing concerns for researchers. The most frequently observed side effects in clinical trials are cytokine-release syndrome (CRS) and CRS-related encephalopathy syndrome (CRES), also named immune effector cell-associated neurotoxicity syndrome (ICANS). The supraphysiologic stimulation caused by CAR molecules with high affinity for antigens may lead to the overproduction of cytokines, such as IFN-g, IL-2, TNF-a, MIP-1, and GM-CSF (44). Moreover, it may elevate other proinflammatory cytokines, including IL-6, IL-8 and IL-10, generated by other bystander immune cells, resulting in even more severe hyperactive immune disorders, hemophagocytic lymphohistiocytosis (HLH) and macrophage activation syndrome (MAS) (45). To cope with these life-threatening side effects, the current strategy includes pharmacological interventions, such as anti-IL-6R mAb (tocilizumab), anti-IL-6 mAb (siltuximab) or corticosteroids, and supportive care depending on the CRS grade (46). Nevertheless, as it would be unwise to wait for the appearance of CRS, priority was given to preventative technologies, for example, establishing a better predictive system based on valid biomarkers (47), improving the “safer CAR” construct with “suicide switch” or “remote-controlled switch” (48, 49), and preventing side effects prophylactic drug administration (50), all of which require further confirmation in clinical trials.

The clinical breakthroughs of CAR-based immunotherapy in hematopoietic cancers have not been duplicated in solid tumors. Several core problems remain unsolved, including tumor-associated antigen (TAA) heterogeneity, restriction of immune cell trafficking and infiltration and an immune-suppressive microenvironment (51). New strategies have been explored to cope with these challenges. Next-generation sequencing technologies, including immunoproteomics, DNA/RNA sequencing and whole-exome screening to identify somatic mutations in tumor cells, have helped researchers discover the neoantigens or neoepitopes used as TAAs (52, 53). However, TAAs are often shared with normal tissue. The high affinity of CARs that rely on scFv could be dangerous when the vital healthy tissues become the attacked targets even with low antigen expression (54). Moreover, stronger affinity of the CARs demonstrated increased anti-tumor efficacy, but may well result in on-target off-tumor toxicity (55). The balance between the anti-tumor functions of CAR-engineering cells and safety is under active study (56). Of note, our results have distinguished that B7-H3, also known as CD276, might be a promising therapeutic target for CAR-based therapy, and there is evidence that it carries no risk of on-target off-tumor toxicity (57, 58). Recently, our research team also found that SAHA, a pan histone deacetylase inhibitor, could enhance B7-H3.CAR-T cells in solid tumors (59). Elsewhere, novel approaches have been investigated to overcome biological barriers in solid tumors, for example, using local delivery systems; applying anti-vasculature agents, chemokines or oncolytic viruses; and equipping effector immune cells with the ability to generate chemokines or heparinase to degrade the extracellular matrix (60). The most difficult obstacle lies in the tumor microenvironment (TME). The harsh physical conditions (hypoxic, poorly vascularized and with excessive interstitial pressure), immune-suppressive cell components [myeloid-derived suppressor cells (MDSCs), tumor-associated macrophages (TAMs), regulatory T cells (Tregs), etc.] and abnormal metabolism caused by nutrient deprivation create a hostile environment for effector cells, leading to their impaired persistence and terminal exhaustion (61). Four major lines of the researches related to this issue are under examination: combination therapies with exogenous antagonists or cytokines, removal of specific immunosuppressive factors in effector cells, modification of CAR structure to avoid immune suppression, and the recently discovered CAR exosomes derived from CAR-T cells which displayed satisfying cytotoxic capability without PD-1 expression (51, 60–63).

In addition to T lymphocytes, recently, researchers have focused on CAR-engineered innate or innate-like immune cells, including NK cells, macrophages, dendritic cells, NKT cells and γδT cells (12, 13). Of note, CAR-NK therapy has made progress in both liquid cancers and solid tumors (12, 64, 65). First, the superior safety of CAR-NK therapy was evidenced by the low incidences of CRS, neurotoxicity and GVHD and relatively low on-target off tumor toxicity (66). Second, CAR NK cells can kill tumor cells through CAR-dependent and NK cell receptor-dependent mechanisms, such as inducing apoptosis of target cells by releasing TNF-α, inducing ADCC mediated by CD16, and activating other immune cells by producing IFN-γ, which may kill off-target tumor cells (67). The most appealing advantage is the potential to provide “off-the-shelf” CAR-engineered products generated from the NK-92 cell line, peripheral blood mononuclear cells (PBMCs), umbilical cord blood (UCB), and induced pluripotent stem cells (iPSCs) (14, 68). Further studies should focus on enhancing the tumor infiltrating ability, extending the memory of short-lived NK cells, and optimizing the manufacturing procedure of these products (12, 24, 68).

The improvements discussed above are greatly supported by the rapid improvement of CAR modification technologies. Thanks to the development of gene editing techniques, from retroviral vector-mediated or lentiviral vector-mediated gene transfer to nonviral methods for gene engineering, many of the problems mentioned above may be solved in the near future (69). The NOT gate (70) or AND gate (71) design of dual CAR constructs protects normal tissues from CAR-T cells, while OR gate designs, including bicistronic CAR (72) and tandem CAR (73), enhance the detection capability of tumor-specific T cells to prevent tumor escape. Using transcription activator–like effector nucleases (TALENs) to disrupt the expression of the panlymphocyte molecule CD52 and the αβ T cell receptor (TCRαβ) in CAR-T cells, Qasim et al. generated the universal CAR (74), which represented a step forward to the off-the-shelf, allogeneic CAR-modified products. Moreover, the Nobel prize awarding CRISPR–Cas9 technology also plays an important role in CAR editing, such as knocking out the PD-1 gene to avoid suppressive signaling in the TME (75). Unlike other randomly integrating vectors, the CRISPR–Cas9 system could conduct efficient sequence-specific interventions, such as directing a CD19-specific CAR to the T-cell receptor α constant (TRAC) locus to generate universal CAR-T cells (76). This system could also be adopted in modifying other immune cells, such as NK cells or macrophages, to knock out immune checkpoints or enhance the expression of stimulatory cytokines, activating signals or homing receptors (68, 75, 77).

The major limitations of the CAR-based immunotherapy and the corresponding potential strategies discussed above are summarized in **Table S4**. Although we conducted a relatively thorough analysis in this study, its limitations should not be neglected. First, the included studies were limited to articles and reviews written in English and recorded in the WoSCC database, which may exclude some valuable studies, but we believe that this would not affect the general trend substantially, as systematic reviews have reported similar findings. Second, patent information was not included in this study and may need further comprehensive analysis. Finally, even with three different analytical tools, it is difficult to completely avoid bias introduced by machine algorithms.

## Conclusion

In summary, a bibliometric analysis of CAR-based immunotherapy in cancer from 2001 to 2021 was performed through automatic analysis software. We found an increasing interest in this field worldwide, with the US being the leading country with the most publications and China being one of the most active major participants. The collaboration networks among institutions and among authors were close and comprehensive. The cutting-edge directions and hotspots in the field are multiple myeloma, safety and toxicity, solid tumors, CAR-engineered immune cells beyond T cells, and gene editing. We expect safer and more effective CAR-engineered products to be introduced to clinical application in the near future, bringing hope for patients with advanced cancers.

## Data Availability Statement

The raw data supporting the conclusions of this article will be made available by the authors, without undue reservation.

## Author Contributions

ZO, LQ, SF, and JL designed the study. ZO and LQ conducted the literatures searching, data extraction and re-examination. ZO, HR, TL, HL, QL, FW, TC, and YY performed the bibiometric analysis. ZO, BL, SR, SK, and LY performed the visualization. ZO wrote the manuscript. LQ, SF, and JL reviewed and revised the manuscript. All authors contributed to the article and approved the submitted version.

## Funding

This study was supported by grants from the National Natural Science Foundation of China (grant Nos. 81672676, 81772890, 81872194 and 82072990), Guangdong Science and Technology Development Fund (grant Nos. 2016A030313352, 2017A030311011 and 2114050000501), Science and Technology Program of Guangzhou (grant Nos. 201607010108, 201803010060 and 20210201040207), Fundamental Research Funds for the Central Universities (grant Nos. 16ykpy10 and 19ykzd20), National Clinical Key Specialty Construction Project for Department of Oral and Maxillofacial Surgery and The Key Laboratory of Malignant Tumor Gene Regulation and Target Therapy of Guangdong Higher Education Institutes, Sun Yat-sen University (grant No. KLB09001).

## Conflict of Interest

The authors declare that the research was conducted in the absence of any commercial or financial relationships that could be construed as a potential conflict of interest.

## Publisher’s Note

All claims expressed in this article are solely those of the authors and do not necessarily represent those of their affiliated organizations, or those of the publisher, the editors and the reviewers. Any product that may be evaluated in this article, or claim that may be made by its manufacturer, is not guaranteed or endorsed by the publisher.
