# Supplementary material for: Bibliometric Analysis of Chimeric Antigen Receptor-Based Immunotherapy in Cancers From 2001 to 2021
Source: Front Immunol. 2022 Mar 30;13:822004. doi: 10.3389/fimmu.2022.822004 (PMC9005877; doi:10.3389/fimmu.2022.822004)
Supplement: Supplementary file 1 [file DataSheet_1.pdf]

Supplementary Material

Supplementary Figures

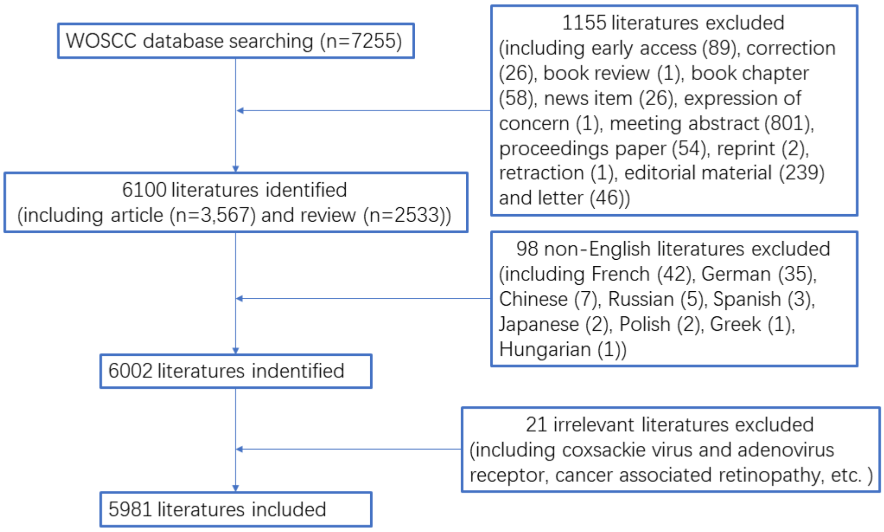

Supplementary Figure 1. The flowchart of the screening procedure.

A

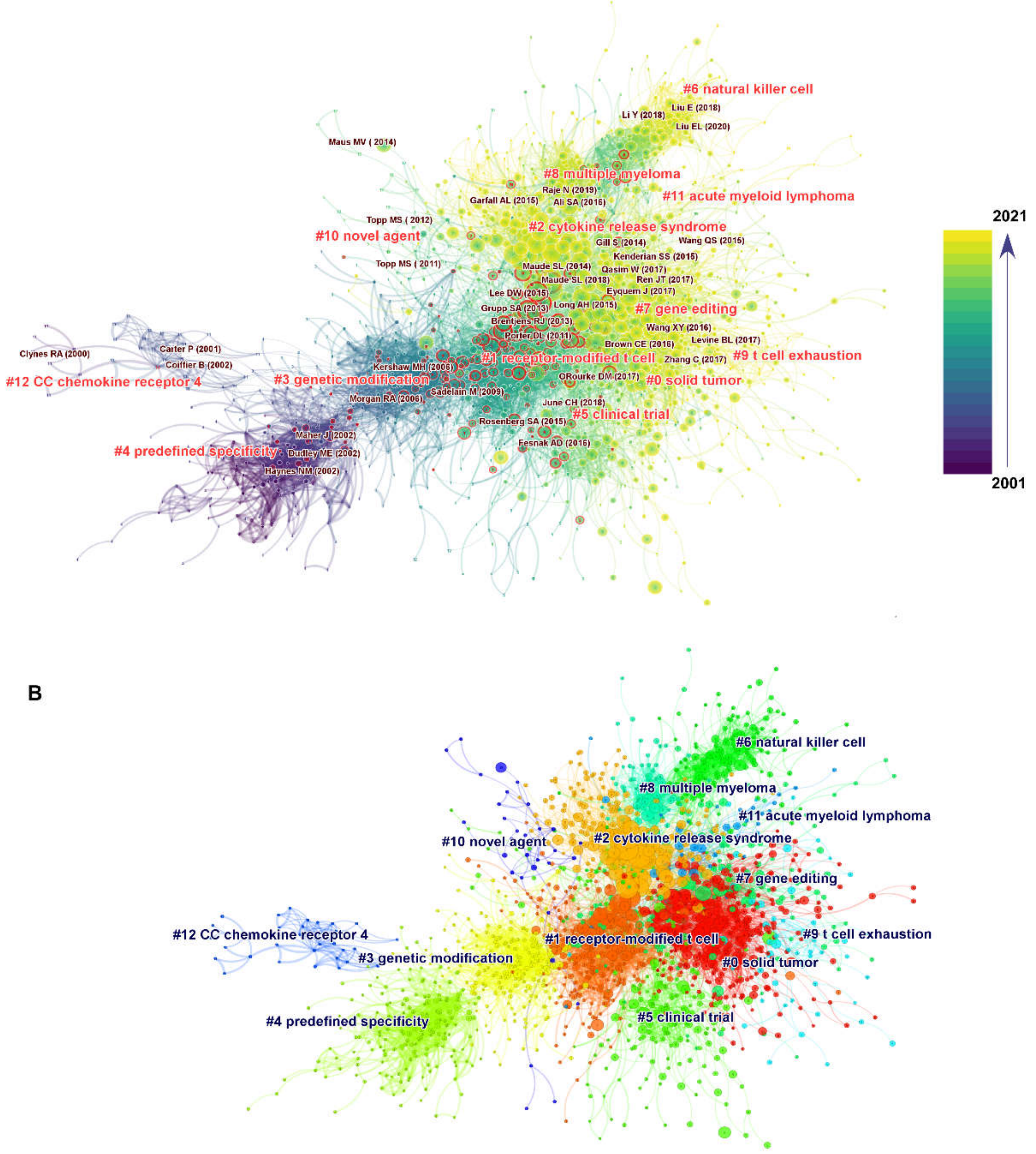

Supplementary Figure 2. The knowledge maps of co-cited references. Each node represents an article or review, and each line represents the link between two studies. **(A)** The time-overlay view of the knowledge map of co-cited references. The size of each node represents the number of co-citations, and the red circle around the node represents the bursts of the references. The tag of each cluster is displayed in the figure. The top 3 co-cited references of each cluster are also displayed above the nodes. **(B)** Cluster view of the knowledge map of co-cited references. The tag of each cluster is also displayed in the figure.

Top 25 References with the Strongest Citation Bursts

References	Year	Strength	Begin	End	2001 - 2021
Pule MA, 2008, NAT MED, V14, P1264, DOI 10.1038/nm.1882, DOI	2008	83.41	**2009**	2013	
Milone MC, 2009, MOL THER, V17, P1453, DOI 10.1038/mt.2009.83, DOI	2009	77.23	**2009**	2014	
Till BG, 2008, BLOOD, V112, P2261, DOI 10.1182/blood-2007-12-128843, DOI	2008	74.61	**2009**	2013	
Carpenito C, 2009, P NATL ACAD SCI USA, V106, P3360, DOI 10.1073/pnas.0813101106, DOI	2009	68.37	**2009**	2014	
Johnson LA, 2009, BLOOD, V114, P535, DOI 10.1182/blood-2009-03-211714, DOI	2009	60.69	**2009**	2014	
Morgan RA, 2010, MOL THER, V18, P843, DOI 10.1038/mt.2010.24, DOI	2010	129.08	**2010**	2015	
Brentjens R, 2010, MOL THER, V18, P666, DOI 10.1038/mt.2010.31, DOI	2010	57.35	**2010**	2015	
Kalos M, 2011, SCI TRANSL MED, V3, P0, DOI 10.1126/scitranslmed.3002842, DOI	2011	185.42	**2011**	2016	
Savoldo B, 2011, J CLIN INVEST, V121, P1822, DOI 10.1172/JCI46110, DOI	2011	114.69	**2011**	2016	
Kochenderfer JN, 2010, BLOOD, V116, P4099, DOI 10.1182/blood-2010-04-281931, DOI	2010	99.48	**2011**	2015	
Robbins PF, 2011, J CLIN ONCOL, V29, P917, DOI 10.1200/JCO.2010.32.2537, DOI	2011	71.08	**2011**	2016	
Porter DL, 2011, NEW ENGL J MED, V365, P725, DOI 10.1056/NEJMoa1103849, DOI	2011	229.57	**2012**	2016	
Kochenderfer JN, 2012, BLOOD, V119, P2709, DOI 10.1182/blood-2011-10-384388, DOI	2012	155.1	**2012**	2017	
Brentjens RJ, 2011, BLOOD, V118, P4817, DOI 10.1182/blood-2011-04-348540, DOI	2011	125.11	**2012**	2016	
Di Stasi A, 2011, NEW ENGL J MED, V365, P1673, DOI 10.1056/NEJMoa1106152, DOI	2011	91.77	**2012**	2016	
Louis CU, 2011, BLOOD, V118, P6050, DOI 10.1182/blood-2011-05-354449, DOI	2011	74.64	**2012**	2016	
Till BG, 2012, BLOOD, V119, P3940, DOI 10.1182/blood-2011-10-387969, DOI	2012	60.59	**2012**	2017	
Brentjens RJ, 2013, SCI TRANSL MED, V5, P0, DOI 10.1126/scitranslmed.3005930, DOI	2013	156.55	**2013**	2018	
Topalian SL, 2012, NEW ENGL J MED, V366, P2443, DOI 10.1056/NEJMoa1200690, DOI	2012	55.37	**2013**	2017	
Grupp SA, 2013, NEW ENGL J MED, V368, P1509, DOI 10.1056/NEJMoa1215134, DOI	2013	196.96	**2014**	2018	
Sadelain M, 2013, CANCER DISCOV, V3, P388, DOI 10.1158/2159-8290.CD-12-0548, DOI	2013	72.07	**2014**	2018	
Kloss CC, 2013, NAT BIOTECHNOL, V31, P71, DOI 10.1038/nbt.2459, DOI	2013	62.76	**2014**	2018	
Maude SL, 2014, NEW ENGL J MED, V371, P1507, DOI 10.1056/NEJMoa1407222, DOI	2014	190.9	**2015**	2019	
Davila ML, 2014, SCI TRANSL MED, V6, P0, DOI 10.1126/scitranslmed.3008226, DOI	2014	116.2	**2015**	2019	
Lee DW, 2014, BLOOD, V124, P188, DOI 10.1182/blood-2014-05-552729, DOI	2014	58.2	**2015**	2019	

Supplementary Figure 3. The top 25 references with strongest citation bursts.

Supplementary Tables

Table S1. Top 10 co-cited references related to CAR-based immunotherapy in hematopoietic malignances.

Rank	Reference	Citations	Author	Year	Type	Journal	IF	JCR
1	Chimeric antigen receptor t cells for sustained remissions in leukemia	2952	Maude, Shannon L	2014	Article (CT)	The New England Journal of Medicine	91.245	Q1
2	Chimeric antigen receptor–modified T cells for acute lymphoid leukemia	2195	Grupp, Stephan A	2013	Article (CT)	The New England Journal of Medicine	91.245	Q1
3	Axicabtagene Ciloleucel CAR T-cell therapy in refractory large B-cell lymphoma	1960	Neelapu, Sattva S	2017	Article (CT)	The New England Journal of Medicine	91.245	Q1
4	Tisagenlecleucel in children and young adults with B-cell lymphoblastic leukemia	1886	Maude, Shannon L	2018	Article (CT)	The New England Journal of Medicine	91.245	Q1
5	T cells expressing CD19 chimeric antigen receptors for acute lymphoblastic leukaemia in children and young adults: a phase 1 dose-escalation trial	1699	Lee, Daniel W	2015	Article (CT)	LANCET	79.321	Q1
6	Efficacy and toxicity management of 19-28z CAR T cell therapy in B cell acute lymphoblastic leukemia	1548	Davila, Marco L	2014	Article (CT)	Science Translational Medicine	17.956	Q1
7	CD19-targeted T cells rapidly induce molecular remissions in adults with chemotherapy-refractory acute lymphoblastic leukemia	1342	Brentjens, Renier J	2013	Article (CT)	Science Translational Medicine	17.956	Q1
8	Chemotherapy-refractory diffuse large B-cell lymphoma and indolent B-cell malignancies can be effectively treated with autologous T cells expressing an anti-CD19 chimeric antigen receptor	1028	Kochenderfer, James N	2015	Article (CT)	Journal of Clinical Oncology	44.544	Q1
9	CD19 CAR–T cells of defined CD4+:CD8+ composition in adult B cell ALL patients	1001	Turtle, Cameron J	2016	Article (CT)	Journal of Clinical Investigation	14.808	Q1
10	Chimeric antigen receptor T cells persist and induce sustained remissions in relapsed refractory chronic lymphocytic leukemia	989	Porter, David L	2015	Article (CT)	Science Translational Medicine	17.956	Q1

CT, clinical trial; IF, impact factors; JCR, journal citation reports; Citation data was updated to 21st February, 2022.

Table S2. Top 10 co-cited references related to CAR-based immunotherapy in solid tumors.

Rank	Reference	Citations	Author	Year	Type	Journal	IF	JCR
1	Case report of a serious adverse event following the administration of T cells transduced with a chimeric antigen receptor recognizing ErbB2	1479	Morgan, Richard A	2010	Article (CR)	Molecular Therapy	11.454	Q1
2	Tumor regression in patients with metastatic synovial cell sarcoma and melanoma using genetically engineered lymphocytes reactive with NY-ESO-1	1049	Robbins, Paul F	2011	Article (CT)	Journal of Clinical Oncology	44.544	Q1
3	Antitumor activity and long-term fate of chimeric antigen receptor–positive T cells in patients with neuroblastoma	846	Louis, Chrystal U	2016	Article (CT)	Blood	22.113	Q1
4	Regression of Glioblastoma after Chimeric Antigen Receptor T-Cell Therapy	742	Brown, Christine E	2016	Article (CR)	The New England Journal of Medicine	91.245	Q1
5	Virus-specific T cells engineered to coexpress tumor-specific receptors: persistence and antitumor activity in individuals with neuroblastoma	710	Pule, Martin A	2008	Article (CT)	Nature Medicine	53.440	Q1
6	A single dose of peripherally infused EGFRvIII-directed CAR T cells mediates antigen loss and induces adaptive resistance in patients with recurrent glioblastoma	646	O'Rourke, Donald M	2017	Article (CT)	Science Translational Medicine	17.956	Q1
7	Human epidermal growth factor receptor 2 (HER2) –specific chimeric antigen receptor–modified T cells for the immunotherapy of HER2-positive sarcoma	542	Ahmed, Nabil	2015	Article (CT)	Journal of Clinical Oncology	44.544	Q1
8	Combinatorial antigen recognition with balanced signaling promotes selective tumor eradication by engineered T cells	530	Kloss, Christopher C	2013	Article	Nature Biotechnology	54.908	Q1
9	Mesothelin-specific chimeric antigen receptor mRNA-engineered T cells induce antitumor activity in solid malignancies	499	Beatty, Gregory L.	2014	Article (CR)	Cancer Immunology Research	11.151	Q2
10	Human CAR T cells with cell-intrinsic PD-1 checkpoint blockade resist tumor-mediated inhibition	457	Leonid, Cherkassky	2016	Article	Journal of Clinical Investigation	14.808	Q1

CT, clinical trial; CR, case report; IF, impact factors; JCR, journal citation reports; Citation data was updated to 21st February, 2022.

Table S3. Summary of target antigens in CAR-based immunotherapy.

Target antigen	CAR-based cell type	Cancer type	Specific cancer type	Occurrence	Total link strength	Highest level of clinical trial phase
CD19	T/NK/iNKT/ γδT	liquid	ALL, BCL, BL, CLL, DLBCL, FL, Mantle cell and follicular lymphoma, MALT, PMBCL	419	4807	FDA approved
CD20	T	liquid	Relapsed and refractory BCL, CLL, DLBCL, FL, MCL, NHL	88	1111	1/2
BCMA	T/NK	liquid	MM	67	826	1/2
CD38	T	liquid	AML, Relapsed or refractory MM	41	492	1/2
CD30	T	liquid	HL	28	345	1/2
CD123	T/NK	liquid	AML	27	336	1/2
CD22	T/NK	liquid	Refractory BCL, Relapsed or refractory DLBCL, B-ALL	26	313	Early 1
CD33	T/NK	liquid	AML	21	240	1/2
CD5	T/NK	liquid	T-cell ALL, T-cell NHL	16	190	1/2
CS1	T/NK	liquid	Recurrent or refractory PCM, Relapsed or Refractory MM	11	122	1
CD138	T	liquid	Relapsed or refractory MM	7	80	1
CD7	T/NK	liquid	ALL, T-cell leukemia, lymphoma	4	36	1/2
ROR1	T	liquid/ solid	Recurrent ALL, Recurrent MCL, Refractory CLL, NSCLC, Breast cancer	18	183	1
CD70	T	liquid/ solid	AML, NHL, MM, Pancreatic cancer, Renal cell cancer, Breast cancer, Melanoma, Ovarian cancer	6	57	1/2
HER2	T/NK	solid	Glioblastoma, Colorectal cancer, Sarcoma, Biliary tract cancer, Pancreatic cancer	113	1423	1/2
Mesothelin	T/NK	solid	Ovarian cancer, Pleural mesothelioma, Pancreatic cancer	103	1211	Early 1
NKG2D ligands	NK/γδT	solid	Solid tumors	94	1163	1
GD2	T/iNKT	solid	Neuroblastoma, Melanoma	69	868	1
EGFR	T	solid	NSCLC	62	694	1
MUC1	T/NK	solid	NSCLC, Hepatic cancer, Pancreas cancer, Breast cancer, Colon cancer, Gastric cancer	46	570	1/2
EGFRvIII	T	solid	Glioblastoma	35	434	1
B7-H3	T	solid	Pediatric solid tumor, Ovarian cancer, Neuroblastoma, Diffuse intrinsic pontine glioma, Diffuse midline glioma, Recurrent and refractory pediatric central nervous system tumors, Recurrent and refractory glioblastoma	32	324	1/2
Folate receptor-α	T	solid	Ovarian cancer	28	401	1
CEA	T	solid	Colorectal cancer, Gastro-esophageal cancer, Pseudomyxoma peritonei, Pancreatic cancer, Liver	25	299	1

			metastases			
c-MET	T	solid	Breast cancer	16	172	0
NY-ESO-1	T	solid	Esophagus cancer	10	134	1/2
PSMA	T/NK	solid	Prostate cancer	10	131	1
CD44v6	T	solid	CD44v6 positive cancers	6	77	1/2
GPC3	T/NK	solid	Colorectal cancer, Hepatocellular Carcinoma	6	64	1
AXL	T	solid	Liver Cancer, Lung Cancer, Breast Cancer, Colorectal Cancer, Brain Tumor	4	45	1
IL13Ra2	T	solid	Glioblastoma	3	41	Case report
FAP	T	solid	Malignant pleural mesothelioma	3	30	1
CD171	T	solid	Neuroblastoma	2	28	1
TAG-72	T	solid	Colorectal cancer	2	27	1
Lewis-Y	T	solid	Liver Cancer, Lung cancer, Breast cancer, Colorectal cancer, Brain tumor	1	15	1
CAIX	T	solid	Renal cell carcinoma	1	13	1

AML, acute myelocytic leukemia; B-ALL, B cell acute lymphoblastic leukemia; BCL, B-cell lymphoma; BL, Burkitt's lymphoma; CLL, chronic lymphocytic leukemia; DLBCL, diffuse large B cell lymphoma; FL, follicular lymphoma; HL, Hodgkin lymphoma; MALT, mucosa-associated lymphoid tissue; MM, multiple myeloma; NHL, non-Hodgkin lymphoma; MCL, mantle cell lymphoma; NSCLC, non–small cell lung cancer; PCM, plasma cell myeloma; PMBCL, primary mediastinal B cell lymphoma.

Table S4. The major limitations of CAR-based immunotherapy and the potential strategies.

Limitations	Potential strategies
Antigen heterogeneity	Next-generation sequencing technologies to discover the neoantigens or neoepitopes
Antigen escape	Sequential use of different CAR-based products
	Multi-CAR constructs (OR gate)
	CAR-BiTE constructs
TME of solid tumors	Overcome the biological barriers
	Local delivery systems
	Anti-vasculature agents, chemokines or oncolytic viruses
	Facilitate CAR-based products to generate chemokines or heparinase
	Combination therapies with exogenous antagonists or cytokines
	Removal of specific immunosuppressive factors in effector cells (MDSCs depletion)
	Metabolic reprogramming
	Modification of CAR structure to avoid immune-suppression
T cell exhaustion	Combination of checkpoint inhibitor with CAR-T (anti-PD-1/PD-L1 mAb)
	PD-1 knockout autologous CAR T cells
	CAR-exosomes derived from CAR-T cells
Toxicity (CRS, CRES, HLH, MAS)	Pharmacological interventions
	Blockade of IL receptor (anti-IL-6R mAb)
	Neutralization of cytokines (anti-IL-6 mAb)
	Corticosteroids
	Supportive care
	Predictive system based on valid biomarkers
	Prophylactic drug administration
	"Safer CAR" construct
	iCasp9 "suicide switch"
	Tet-On inducible "remote-controlled switch"
	Multi-CAR constructs (NOT or AND gate)
	Tuning CAR affinity
Financial burden	Off-the-shelf CAR-based products
	Novel gene engineering techniques
	CRISPR–Cas9
	CAR-T cell produced in vivo through mRNA/LNP system

BiTE, bispecific T-cell engager; TME, tumor microenvironment; CRS, cytokine-release syndrome; CRES, CRS-related encephalopathy syndrome; HLH, hemophagocytic lymphohistiocytosis; MAS, macrophage activation syndrome; LNP, Lipid nanoparticles.
